# Supplementary material for: Yellow Sticky Cards Reduce the Numbers of Trichogramma dendrolimi (Hymenoptera: Trichogrammatidae) Following Augmentative Releases against the Fruit Borers Carposina sasakii (Lepidoptera: Carposinidae) and Grapholita molesta (Lepidoptera: Tortricidae) in a Pear Orchard
Source: Insects. 2024 Aug 3;15(8):590. doi: 10.3390/insects15080590 (PMC11354991; doi:10.3390/insects15080590)
Supplement: Supplementary file 1 [file insects-15-00590-s001.zip › insects-3065106-supplementary.pdf]

Supplementary File

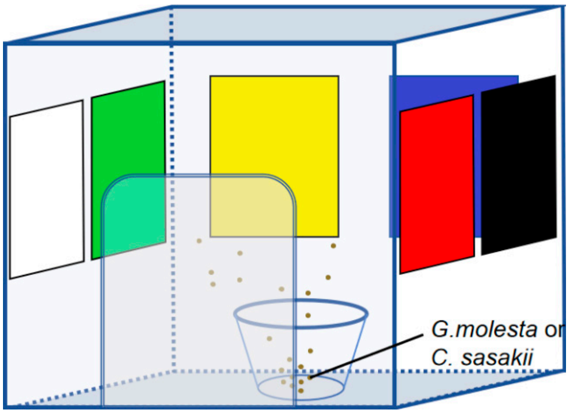

Figure S1. Installation diagram of the test for *C. sasakii* and *G. molesta* preferences for different colored sticky boards.

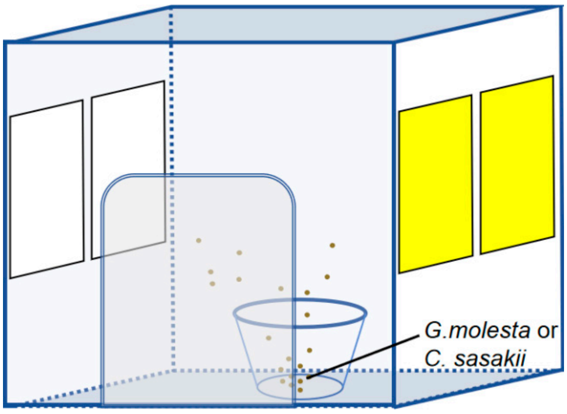

Figure S2. Installation diagram of the preferential selection, by *C. sasakii* and *G. molesta*, between yellow and white sticky boards.
